# Vapor-Phase Hydrogenation of Levulinic Acid to γ-Valerolactone Over Bi-Functional Ni/HZSM-5 Catalyst

**DOI:** 10.3389/fchem.2018.00285

**Published:** 2018-07-17

**Authors:** Margarita Popova, Petar Djinović, Alenka Ristić, Hristina Lazarova, Goran Dražić, Albin Pintar, Alina M. Balu, Nataša Novak Tušar

**Affiliations:** ^1^Institute of Organic Chemistry with Centre of Phytochemistry, Bulgarian Academy of Sciences, Sofia, Bulgaria; ^2^National Institute of Chemistry, Ljubljana, Slovenia; ^3^Departamento de Quimica Organica, Universidad de Cordoba, Córdoba, Spain; ^4^University of Nova Gorica, Nova Gorica, Slovenia

**Keywords:** Ni/HZSM-5, Ni/Al molar ratio acidity regulation, vapor-phase hydrogenation, levulinic acid conversion, γ-valerolactone selectivity, biomass valorization

## Abstract

The hydrogenation of levulinic acid (LA) to γ-valerolactone (GVL) in vapor-phase is economically more viable route if compared to reaction in liquid-phase. To improve the GVL yield in the vapor-phase reaction, the optimization of nickel modified zeolite as bi-functional catalyst (Ni/HZSM-5) was studied. Ni/HZSM-5 materials with fixed Al/Si molar ratio of 0.04 and different nominal Ni/Si molar ratios (from 0.01 to 0.05) were synthesized without the use of organic template and with the most affordable sources of silica and alumina. Materials were characterized by X-ray powder diffraction, SEM-EDX, TEM-EDX, pyridine TPD and DRIFTS, H_2_-TPR, N_2_ physisorption and isoelectric point. In the synthesized materials, 61–83% of nickel is present as bulk NiO and increases with nickel content. Additionally, in all catalysts, a small fraction of Ni^2+^ which strongly interacts with the zeolite support was detected (10–18%), as well as Ni^2+^ acting as charge compensating cations for Brønsted acid sites (7–21%). Increasing the nickel content in the catalysts leads to a progressive decrease of Brønsted acid sites (BAS) and concomitant increase of Lewis acid sites (LAS). When BAS/LAS is approaching to 1 and at the same time the amount of NiO reducible active sites is around 80%, the bi-functional Ni/HZSM-5-3 catalyst (Ni/Al = 0.59) leads to 99% conversion of LA and 100% selectivity to GVL at 320°C. This catalyst also shows stable levulinic acid hydrogenation to GVL in 3 reaction cycles conducted at 320°C. The concerted action of the following active sites in the catalyst is a key element for its optimized performance: (1) Ni metallic active sites with hydrogenation effect, (2) Lewis acid sites with dehydration effect, and (3) nickel aluminate sites with synergetic and stabilizing effects of all active sites in the catalyst.

## Introduction

The decrease in fossil fuel reserves and the high price of petrochemicals have focused the attention to the renewable energy resources (Corma et al., 2007; Hayes, 2009; Bond et al., 2010; Climent et al., 2014; Mika et al., 2018). Lignocellulosic biomass is a promising inexpensive renewable material that could satisfy society's requirements for chemicals and fuels (Corma et al., 2007; Chang, 2011; Climent et al., 2014; Li et al., 2016). Lignocellulosic biomass can be hydrolyzed into a mixture of cellulose, hemicellulose and lignin. Further hydrolysis of the hemicellulose and cellulose leads to the formation of C5 and C6 monosaccharides. Levulinic acid (LA) can be derived from lignocellulosics via acid-catalyzed hydrolysis processes (Wettstein et al., 2012; Wright and Palkovits, 2012) and can be utilized as a platform molecule for the production of valuable products including biofuels precursors such as γ-valerolactone (GVL) (Son et al., 2014). GVL can be used as a solvent, fuel additive, and intermediate in the production of diverse value-added chemicals (Horváth et al., 2008; Heeres et al., 2009; Serrano-Ruiz and Dumesic, 2011; Kumar et al., 2015; Long et al., 2015; Li et al., 2016; Jiang et al., 2016; Song et al., 2017).

GVL has been produced from LA via catalytic hydrogenation to hydroxyvaleric acid followed by ring closing and dehydration to GVL (Ruiz et al., 2010). In recent years, a lot of research has been done on the catalytic hydrogenation of LA to GVL using homogeneous, as well as heterogeneous catalysts (Shu et al., 1995; Hengne et al., 2012; Yan and Chen, 2014; Abdelrahman et al., 2014; Nadgeri et al., 2014; Song et al., 2017; Sun et al., 2017). However, heterogeneous catalytic processes are more economical as they offer advantages such us easy recovery and recycling. Typically GVL could be produced by liquid-phase hydrogenation of LA using heterogeneous catalytic processes over supported noble metal (Ru, Ir, and Pd), or non-noble metal catalysts such as Co, Cu, and Ni (Shu et al., 1995; Hengne et al., 2012; Nadgeri et al., 2014; Yan and Chen, 2014; Song et al., 2017; Sun et al., 2017). In spite of the excellent performance, unfortunately, some drawbacks including high cost of noble metals, and metal leaching in harsh conditions limit their applications for large-scale LA production. Additionally, the liquid phase conversion of LA to GVL has a number of disadvantages, such as the requirement for high pressure, purification after the reaction process, as well as safety and waste emission. Vapor-phase hydrogenation of levulinic acid gives the opportunity to overcome these disadvantages.

To the best our knowledge, only limited studies were performed from 2015 to 2018 in vapor-phase hydrogenation in which Cu, Co and Ni supported catalysts were studied (Kumar et al., 2015, 2016; Sun et al., 2017; Yoshida et al., 2017; Lomate et al., 2018). The optimization of reaction parameters (temperature, pressure, space velocity, reaction time) and type of used catalysts are key factors for development of technology for preparation of GVL by hydrogenation of levulinic acid (Galletti et al., 2012; Zhou et al., 2014). Another important task is related to the increase of the levulinic acid conversion by the addition of stable solid acid co-catalysts as supports (i.e., zeolites) to conventional catalysts.

Zeolites are well known and widely employed industrial acid catalysts (Ertl et al., 2008). The introduction of low-cost transition metals supported on zeolite is a promising way for industrial applications requiring bi-functional catalysts (metallic and acidic function) to achieve the desired chemical conversions. Zeolites are acidic microporous crystalline aluminosilicates. Among many different zeolite structure types, the MFI (ZSM-5) has been widely reported for its use in the catalytic treatment of hydrocarbons. Recent studies report that sitting and distribution of Al sites in zeolites (Brönsted acid sites) is not statistical, but depends on the zeolite hydrothermal synthesis conditions (Perea et al., 2015). The acidity and textural properties of zeolites can be modified by the addition of transition metal oxides, obtaining bi-functional catalysts. Acidity regulation of bi-functional catalyst balances its hydrocarbon cracking function and can slow down the coke formation, thus contributing to a longer catalyst lifetime. On the basis of our previous experience (Szegedi et al., 2007), nickel metallic species exhibit higher intrinsic activity for toluene hydrogenation reaction and they are generally regarded as the active sites in traditional hydrogenation catalysts. Recently, Ni/HZSM-5 with excellent stability for the cascade transformation of LA to GVL with 100% yield was developed, where acidity was regulated by potassium (Al/Si = 0.026, 10 wt. % Ni and 0.5% K) (Sun et al., 2016).

In the present study, we describe the application of Ni functionalized HZSM-5 catalyst for vapor-phase hydrogenation of levulinic acid to γ-valerolactone with 100% yield, where the catalytic properties of the catalysts are regulated by Ni/Al molar ratio. In this way, the requirement for additional metal to attenuate the acidity of the catalyst was avoided, which makes the synthesis of the catalysts simpler and more economic. A template-free ZSM-5 zeolite was synthesized, which is considered as a green and sustainable process, avoiding the use of any organic structure-directing agents (templates). The synthesis process was developed in collaboration between the National Institute of Chemistry and a local zeolite producer in Slovenia, SILKEM (Fakin et al., 2015). The structural, acidic and catalytic properties of ZSM-5 support with different Al/Si molar ratio were described in our previous study. The ZSM-5 support with optimal Al/Si molar ratio 0.04 was chosen for this study (Ojeda et al., accepted).

## Materials and methods

### Materials

Sodium aluminate, sodium aqueous glass NaVS3M, sulphuric acid, ammonium sulfate and nickel (II) nitrate hexahydrate (Ni(NO_3_)_2_·6H_2_O were provided by Sigma Aldrich. Sodium hydroxide was provided by Merck. ZSM-5 crystallization seeds were provided from Zeolyst (CBU2314, SiO_2_/Al_2_O_3_ = 23).

### Synthesis

ZSM-5 was prepared by seed-assisted synthesis following a procedure previously reported (Fakin et al., 2015). ZSM-5 crystallization seeds (CBU2314, Zeolyst, SiO_2_/Al_2_O_3_ = 23) were dissolved in distilled water (solution A). Sodium aqueous glass (NaVS3M, Silkem, Na_2_O = 8.62 %, SiO_2_ = 27.83 %) was added to solution A during stirring at room temperature with the aqueous solution of sodium aluminate (Silkem, γ(Na_2_O) = 170.19 g/L, γ(Al_2_O_3_) = 148.46)—solution B. Aqueous solution of sulphuric acid (Sigma Aldrich, 96%) was slowly added to the solution B and stirred for 10 min in order to regulate the pH value at 11. Hydrogel was aged for 30 min and then transferred to 1 L reactor (Parr) for crystallization at 180°C for 24 h during continuous stirring. The obtained product was filtered, washed with water, and dried at 60°C overnight. Acidic zeolites were prepared by ${\text{NH}}_{4}^{+}$ exchange. Ammonium sulfate ((NH_4_)_2_SO_4_, Sigma Aldrich, ≥99.0%), distilled water and zeolite (ZSM-5) were used as the main components in the molar ratio of 1:1:20 and mixed at 30°C for 2 h. The exchanged product was filtered, washed with distilled water, dried overnight at 105°C and calcined in air at 500°C for 2 h. The Ni containing HZSM-5 catalysts were obtained by incipient wetness impregnation method using (Ni(NO_3_)_2_·6H_2_O, Sigma Aldrich, purity = 99.999%) with the theoretical molar ratios of Ni/Si = 0.01, 0.03, and 0.05. Finally, the products were calcined in air at 500°C for 2 h, to obtain the corresponding metal oxide (Szegedi et al., 2007). The products were denoted: Ni/HZSM-5-1 (Ni/Si = 0.01), NiHZSM-5-3 (Ni/Si = 0.03), NiHZSM-5-5 (Ni/Si = 0.05).

### Characterization

The prepared catalysts were characterized by different techniques in order to determine their physicochemical properties. The crystal structure of the prepared and spent catalysts was analyzed by X-ray diffraction (XRD) using a PANalytical X'Pert PRO MPD X-ray diffractometer (CuKα_1_ = 0.15406 nm) with an accelerating voltage of 45 kV and an emission current of 40 mA. XRD patterns were obtained at room temperature from 2 Θ from 5 to 70° with a step of 0.034° and time of 16 h.

Scanning electron microscopy (SEM) was used to determine the morphology and particle size of the catalysts using a Zeiss Supra TM 3VP electron microscopy. The elemental analysis was performed by energy-dispersive X-ray spectroscopy (EDXS), using a Zeiss Supra TM 3VP scanning electron microscope with an INCA Energy system attached.

The surface area and pore volume of the samples were determined from N_2_ physisorption isotherms collected at −196°C using a Tristar 3000 analyzer (Micromeritics). The specific surface area was determined using the Brunauer-Emmett-Teller (BET) method. Before N_2_ adsorption, the samples were outgassed under vacuum for 2 h at 200°C in the apparatus. The micropore surface area and micropore volume were determined using the t-plot method.

Isoelectric point, defined as the pH value at which the surface charge is zero, was determined using Zetasizer nano ZS (Malvern) based on electrophoretic scattering of light in the pH range from 2 to 10. The pH value was controlled by a 0.1 M NaOH and 0.1 M HCl solutions. The pH value of each suspension was measured using a digital pH meter.

Thermogravimetric method of pyridine adsorption was used for quantification of acid sites on investigated materials using Pyris 1 TGA apparatus from Perkin Elmer. Prior to analysis, the samples were degassed *in-situ* in N_2_ stream (30 mL/min) at 500°C for 1 h. Afterwards, the samples were cooled to 125°C and saturated with pyridine vapors by passing the N_2_ stream through a saturator filled with liquid pyridine. Saturation was followed by desorption of weakly bound pyridine by degassing the sample in N_2_ at 125°C for additional 2 h until achieving a stable sample weight. Total number of acid sites was calculated based on the weight difference before and after sample saturation. Strength of the acid sites was estimated using temperature programmed desorption based on the assumption that stronger acid sites desorb pyridine at higher temperatures. The sample was heated in N_2_ stream (30 mL/min) until 500°C and mass change was continuously monitored.

Differentiation between Brønsted (BAS) and Lewis (LAS) acid sites was done for pure HZSM-5 and Ni/HZSM-5 materials by DRIFTS analysis using Frontier IR spectrometer (Perkin Elmer), DiffusIR® accessory from Pike Scientific and pyridine as the probe molecule. The powdered samples (~10 mg) were positioned in the ceramic sample cup and pretreated in N_2_ (50 mL/min) at 500°C for 30 min. After cooling to 125°C, the samples were saturated with pyridine vapors (nitrogen (50 mL/min) was bubbled through a saturator filled with liquid pyridine) for 10 min, followed by degassing in vacuum (1 × 10^−5^ mbar) for 1 h. Spectra were recorded with 8 accumulations and spectral resolution of 4 cm^−1^ between 800 and 4,000 cm^−1^. For characterization of LAS and BAS, adsorption bands at 1,445 and 1,545 cm^−1^ were considered. The first originates from pyridine adsorbed on LAS, while the second from pyridinium ion coordinatively bound to BAS. The BAS/LAS ratio was calculated as follows:

$$\begin{array}{l} \text{BAS/LAS}=\text{1.73/1.23}\times {\text{I}}_{\text{BAS}}/{\text{I}}_{\text{LAS}} \end{array}$$

In this equation, I_BAS_ and I_LAS_ represent intensity of absorption bands at 1,545 and 1,445 cm^−1^, 1.73 and 1.23 are extinction coefficients, as reported by Tamura et al. (2012).

Temperature programmed reduction with hydrogen (H_2_-TPR) was used to characterize the interaction between the HZSM-5 support and the supported reducible nickel containing species. The AutoChem II 2920 apparatus from Micromeritics was used. During the experiment, 100 mg of a sample was positioned inside the quartz reactor and pretreated in 20% O_2_/N_2_ at 300°C for 5 min. After sample cooling to 10°C, it was degassed in Ar for 10 min. TPR analysis was performed between 10 and 750°C, 25 mL/min of 5% H_2_/Ar flow and a heating rate of 10°C/min. The recorded TPR curves were deconvoluted with Peakfit software using symmetrical Gaussian peaks to quantitatively evaluate the contribution of NiO, nickel phyllosilicates and Ni^2+^ charge compensating cations.

UV-Vis DR spectra were recorded on a Perkin Elmer Lambda 35 apparatus equipped with a Praying Mantis accessory. Background was recorded with Spectralon® reference. Samples were scanned in the spectral range between 200 and 900 nm, with slit set to 2 nm and scanning speed of 240 nm/min.

Ni/HZSM-5 materials were studied by probe Cs corrected scanning transmission electron microscope Jeol ARM 200 CF with cold-FEG cathode, equipped with dual-EELS (Electron Energy Loss Spectroscopy) system Quantum ER from Gatan and Centurio EDXS system (Energy dispersive X-ray spectroscopy) with 100 mm^2^ SDD detector (Silicon drifted detector). In scanning transmission mode (STEM) two observation techniques were used: high-angle annular dark-field (HAADF) imaging and bright-field (BF) imaging.

### Catalytic activity measurements

Prior to the catalytic tests, samples were pretreated for 1 h in N_2_ flow at 400°C.

Levulinic acid hydrogenation was studied at atmospheric pressure using a fixed-bed flow reactor with hydrogen as carrier gas (30 mL/min). In the reaction, 50 mg sample (particle size 0.2–0.8 mm) was tested, diluted with 50 mg of glass beads of the same diameter, which were previously checked to be inactive. The reactor itself was a quartz tube of 15 mm inner diameter, with the catalyst bed at the middle. A thermocouple was positioned in the catalyst bed for accurate temperature measurements. All gas lines of the apparatus were heated continuously to 150°C in order to minimize condensation of reactants and products on the tube walls. The hydrogen stream passed through a saturator filled with levulinic acid equilibrated at 0°C. The reactants were fed into the reactor with a flow rate of 30 mL/min and catalytic tests were carried out in the temperature range of 250–350°C. The reaction steady state was established after 30 min at each temperature. On-line analysis of the reaction products was performed using HP-GC with a 30 m HP-5MS capillary column. The reusability of the most active sample was studied in 3 reaction cycles and the catalyst was regenerated in air at 500°C, reduced in hydrogen at 400°C, and studied in the catalytic reaction in every reaction cycle.

## Results and discussion

### Physico-chemical characterization

**Figure 1:**
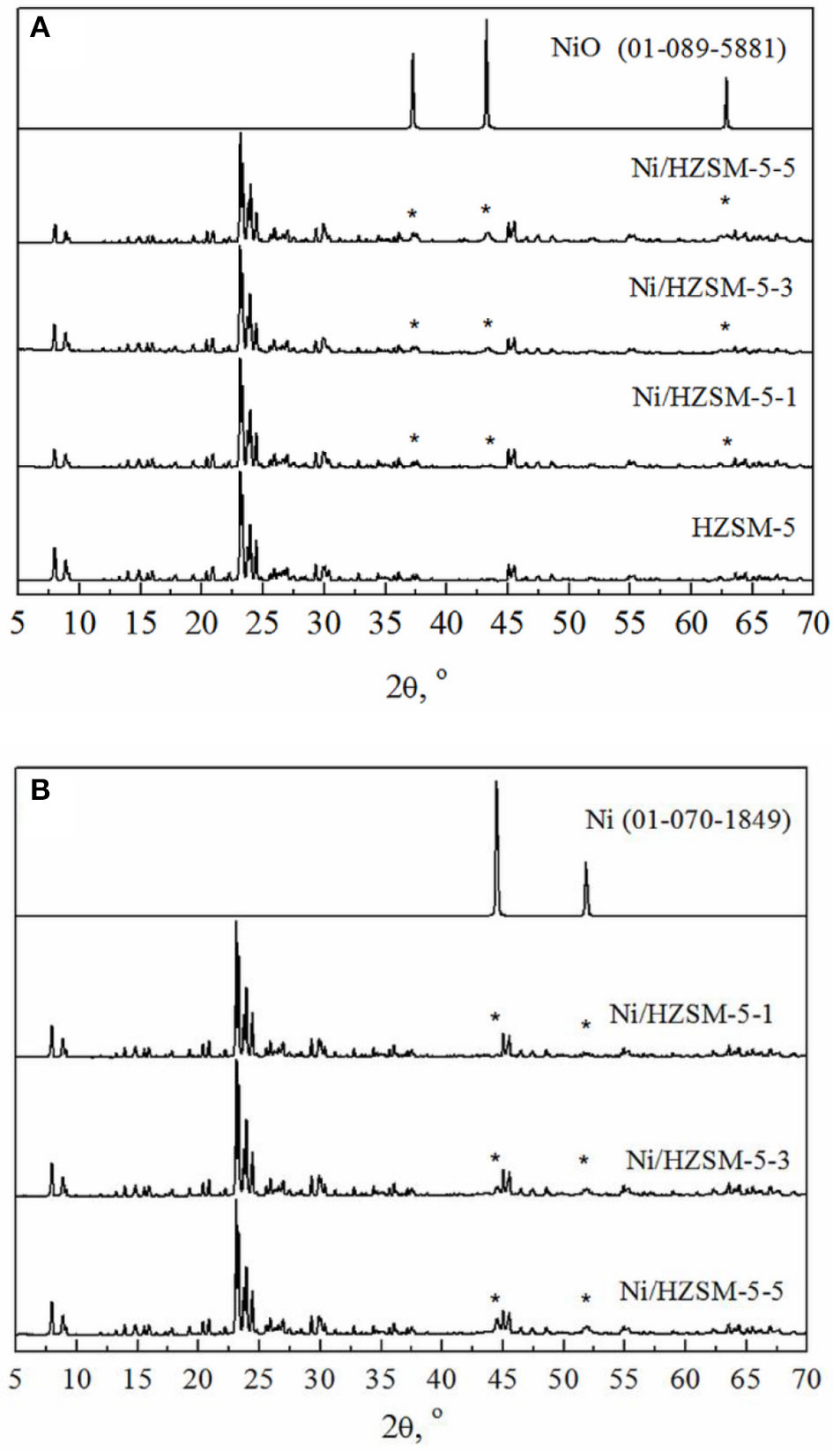
XRD diffractograms of HZSM-5 and Ni/HZSM-5 catalysts before **(A)** and after **(B)** the reaction. Peaks belonging to NiO **(A)** and Ni **(B)** are marked with ^*^.

The X-Ray patterns of the catalysts before the reaction (prepared catalysts) and after the reaction (spent catalysts) are shown in Figures 1A,**B**, respectively. All the prepared catalysts present the typical XRD patterns corresponding to a single crystalline phase with MFI structure. The crystalline structure of HZSM-5 is not affected by deposition of nickel, however, broad diffraction peaks belonging to NiO are detected (Figure 1A), indicating on the formation of nanosized particles, located on the surface of zeolite crystals. The crystallite sizes of NiO are calculated using Scherrer‘s equation based on the selected diffraction peaks of the corresponding XRD pattern and it was found to be 21, 12, and 10 nm for Ni/HZSM-5-5, Ni/HZSM-5-3, and Ni/HZSM-5-1, respectively. No other crystalline phases are detected in the prepared catalysts. XRD patterns (Figure 1B) of the spent catalysts show the preservation of the ZSM-5 structure and the presence of the metallic Ni crystallites, having from 36 (Ni/HZSM-5-3) to 44 nm (Ni/HZSM-5-1 and Ni/HZSM-5-5) in size.

**Figure 2:**
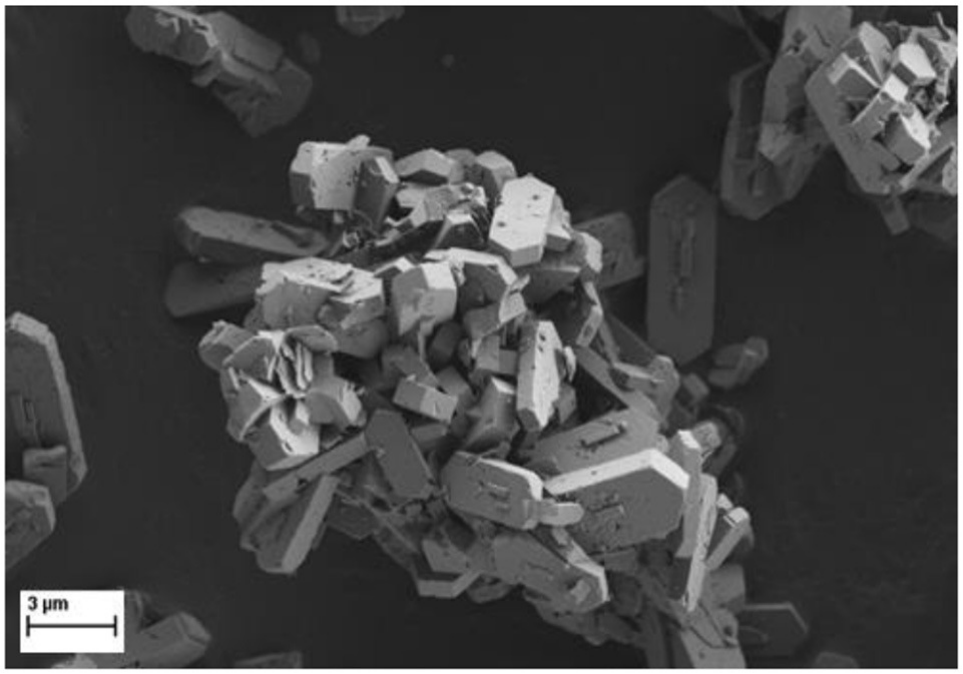
SEM micrograph of Ni/HZSM-5-3 catalyst sample (2.2 wt. % Ni).

SEM photos revealed the presence of nicely formed crystals in all samples. The crystals were about 3–5 μm in size with a typical shape of hexagonal prisms for ZSM-5 (Figure 2).

The data from the EDXS elemental analysis of Ni/HZSM-5 materials are summarized in Table 1. The measured amount of aluminum in the catalysts corresponds closely to the nominal value for all analyzed materials. The measured amount of nickel present in the Ni/HZSM-5-1 and Ni/HZSM-5-3 materials corresponds approximately to the nominal values, whereas it is significantly lower in the Ni/HZSM-5-5 sample.

**Table 1:** Elemental analysis of the Ni/HZSM-5 materials, measured with EDXS technique.

**Catalyst**	**Molar ratio (theoretical)**		**Metal content (EDXS)**		**Molar ratio (practical)**	
	**Ni/Si**	**Al/Si**	**Ni (wt. %)**	**Al (wt. %)**	**Ni/Si**	**Al/Si**
Ni/HZSM-5-1	0.01	0.04	1.18	1.71	0.013	0.042
Ni/HZSM-5-3	0.03	0.04	2.22	1.73	0.025	0.042
Ni/HZSM-5-5	0.05	0.04	2.71	1.71	0.031	0.042

The modification of HZSM-5 by nickel causes the specific surface area of the catalysts to decrease progressively with the increase of nickel content (Table 2). This is most likely due to the deposition of NiO crystallites on the surface of zeolite crystals, which is in agreement with XRD results. Micropore surface area decreased due to Ni^2+^ exchanged Brønsted acid sites in micropores (Ma et al., 2016).

**Table 2:** Structural properties of fresh Ni/HZSM-5 catalysts containing different nickel amounts.

**Catalyst**	**Specific surface area (m^2^/g)**	**Micropore volume (cm^3^/g)**	**Micropore surface area S_mp_ (m^2^/g)**	**External surface area S_ext_ (m^2^/g)**
Ni/HZSM-5-1	400	0.125	313	87
Ni/HZSM-5-3	381	0.119	298	83
Ni/HZSM-5-5	345	0.109	270	75

**Figure 3:**
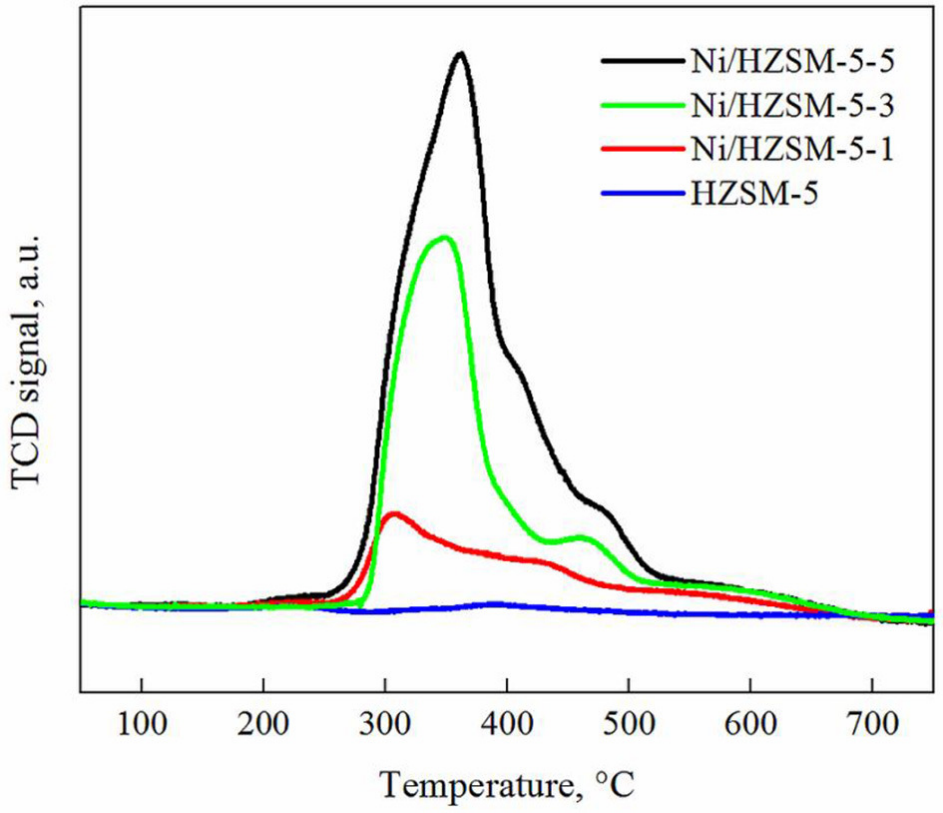
H_2_-TPR profiles of HZSM-5 and Ni/HZSM-5 catalysts containing 1, 2, and 3 weight % of nickel (Ni/HZSM-5-1, Ni/HZSM-5-3, Ni/HZSM-5-5).

The redox properties of the catalysts and interaction between nickel containing reducible phases and HZSM-5 support were analyzed with H_2_-TPR. The reduction profiles of examined Ni/ZSM-5 catalysts (Figure 3) are composed of several overlapping peaks indicating presence of different reducible nickel containing phases. The main envelope of peaks between 300 and 400°C shows a progressive shift to higher temperatures (apex shifts from 306 to 362°C) when the nominal Ni/Si content is increased from 0.01 to 0.05. These peaks can be assigned to reduction of polydisperse bulk NiO having weak or no interaction with the zeolite support (Louis et al., 1993; Yuan et al., 2006; Maia et al., 2010). The NiO crystallites measuring from 8 to 30 nm have been identified performing TEM analysis on all catalysts. The second peak (above 400°C) increases in intensity and its apex shifts toward higher temperatures (from 427 to 477°C) when increasing NiO loading. This is a consequence of increasing amount of Ni^2+^ strongly interacting with the zeolite support. A broad shoulder above 550°C which extends up to 700°C belongs to reduction of Ni^2+^ species, which are distributed over the surface of catalysts as charge compensating cations for Brønsted acid sites (nickel aluminate phase). The H_2_ quantity, consumed during the TPR analyses was sufficient for total reduction of Ni^2+^ to metallic nickel.

The TPR curves were further deconvoluted into several contributions (bulk NiO, Ni^2+^ strongly interacting with the zeolite support and charge compensating Ni^2+^ cations), according to the temperature range where they occur, in order to estimate their contribution to the overall amount of H_2_ consumed. It was found that the bulk NiO is the predominant phase in all analyzed materials, accounting for 61–83% of all nickel. With increasing nickel loading, the fraction of bulk NiO increases due to progressive segregation of this phase on the surface of the zeolite crystals (confirmed by TEM analysis). The Ni^2+^ strongly interacting with the zeolite support accounts for 10–18% of all nickel. The remaining are Ni^2+^ charge compensating cations for the Brønsted acid sites (nickel aluminate phase). Their amount (Table 3) is about an order of magnitude lower compared to the measured total number of Brønsted acid sites in the parent HZSM-5 zeolite (i.e., 0.70 mmol/g_cat_), indicating that nickel addition only slightly decreases their abundance.

**Table 3:** Total H_2_ consumed during TPR analysis and qualitative distribution of different nickel containing species for Ni/HZSM-5 catalysts.

**Sample**	**Total H_2_ consumed, mmol/g_cat_**	**NiO, %**	**Ni^2+^ strongly interacting with the zeolite support, %**	**Ni^2+^ as charge compensating cations, %**
Ni/HZSM-5-1	0.25	61	18	21 (0.042)^a^
Ni/HZSM-5-3	0.57	77	12	11 (0.062)
Ni/HZSM-5-5	0.92	83	10	7 (0.064)

a *Values in parentheses represent the amount of Ni^2+^ (mmol/g_cat_) acting as charge compensating cations for Brønsted acid sites*.

Gravimetric chemisorption of pyridine, followed by TPD was used to evaluate the total number and strength of acid sites present in the catalysts. Based on the amount of chemisorbed pyridine, total acidity changed very little when nickel was deposited to the HZSM-5 support (Table 4). The measured value for the pure HZSM-5 (0.70 mmol/^*^g_cat_) corresponds closely to the theoretical calculation (0.64 mmol/^*^g_cat_), which assumed all Al^3+^ as tetrahedrally coordinated and located in the zeolite framework.

**Table 4:** Total acidity for HZSM-5 and Ni/ZSM-5 samples containing different amounts of nickel and BAS/LAS ratio for the same materials before and after the catalytic reaction.

**Sample**	**Total acidity, mmol/g_cat_**	**Before reaction BAS/LAS, /**	Normalized BAS amount, /^*^	Normalized LAS amount, /^*^	**After reaction BAS/LAS, /**	BAS remaining after reaction, %^**^	LAS remaining after reaction, %^**^
HZSM-5	0.70	13.1	1	0.10	N.D.	N.D.	N.D.
Ni/HZSM-5-1	0.68	1.44	0.78	0.73	2.20	71	48
Ni/HZSM-5-3	0.72	1.03	0.68	0.90	1.41	85	63
Ni/HZSM-5-5	0.75	0.86	0.63	1	1.03	86	73

* *In catalysts before the catalytic reaction*.

** *Calculated as a ratio of characteristic IR peak intensity (1,545 cm^−1^ for BAS and 1,450 cm^−1^ for LAS) after and before reaction*.

**Figure 4:**
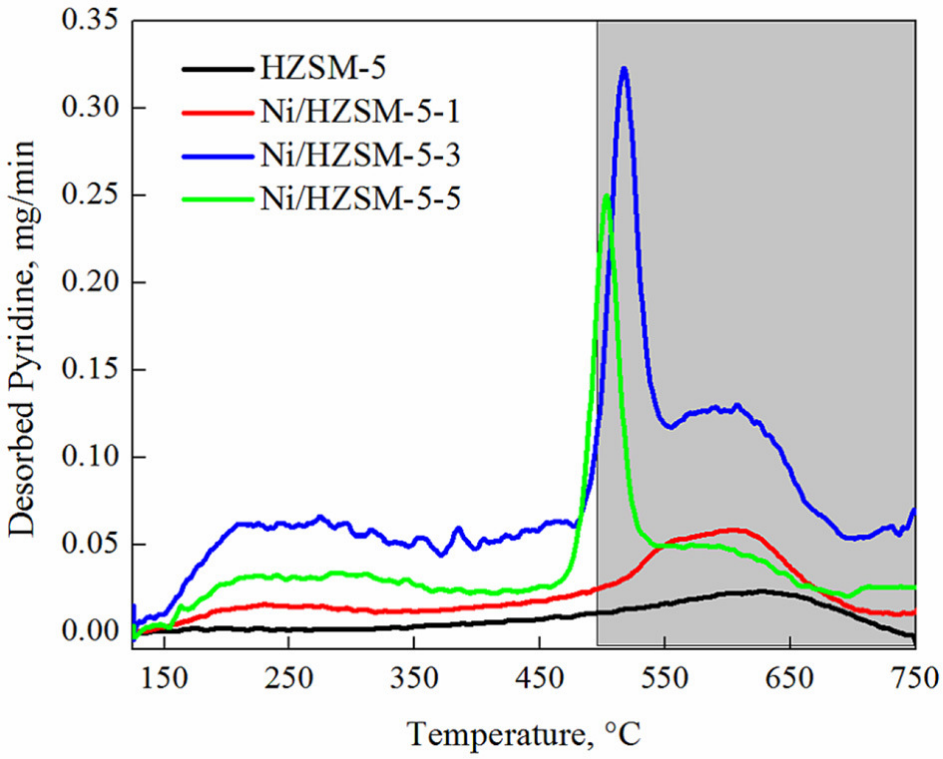
Desorption of pyridine as a function of temperature for Ni/HZSM-5 samples containing 1, 2, and 3 weight % of nickel (Ni/HZSM-5-1, Ni/HZSM-5-3, Ni/HZSM-5-5).

Strength of acid sites was evaluated using temperature programmed desorption of the chemisorbed pyridine. It can be seen in Figure 4 that as nickel content increases, the acid site strength decrease. This is due to the fact that the newly formed Lewis acid sites (LAS - coordinatively unsaturated Ni^2+^), exhibit intrinsically weaker electrophilic character and consequently lower acid site strength compared to Brønsted acid sites. The sharp pyridine desorption peak recorded over Ni/HZSM-5-3 and Ni/HZSM-5-5 catalysts between 470 and 550°C could only tentatively be ascribed to pyridine desorption form the LAS due to the most abundant NiO phase present in these materials. Due to the fact that all samples were pretreated at 500°C (to avoid excessive sintering of nickel containing phases), catalyst dehydroxylation above this temperature (represented by shaded area in Figure 4) could also occur and result in mass loss, which could be erroneously assigned to pyridine desorption.

**Figure 5:**
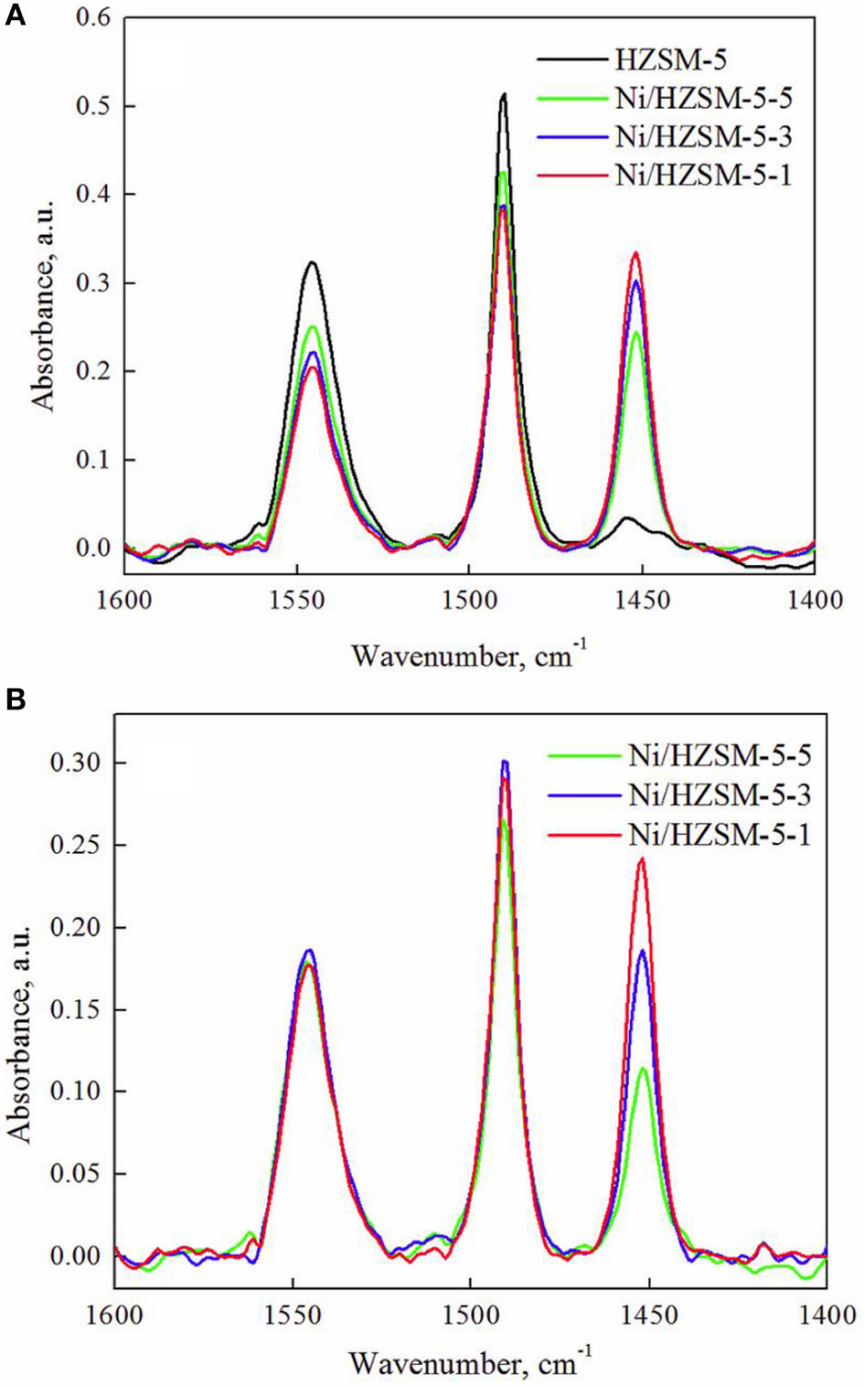
FTIR spectra of pyridine chemisorbed on Brønsted and Lewis acid sites for HZSM-5 and Ni/HZSM-5 samples containing 1, 2, and 3 weight % of nickel (Ni/HZSM-5-1, Ni/HZSM-5-3, Ni/HZSM-5-5): **(A)** before and **(B)** after the catalytic reaction.

Nature of acid sites was analyzed using pyridine as the probe molecule. Pure HZSM-5 contains predominantly Brønsted acid sites (BAS), with BAS/LAS ratio equaling 13.1 (Figure 5A). This confirms the large majority (93 %) of aluminum is present in tetrahedral framework coordination. Addition of increasing nickel amounts leads to a progressive decrease of BAS and concomitant increase of LAS (Table 4), resulting in a BAS/LAS ratio of ~1. The observed changes in the nature of acid sites are due to Ni^2+^ cations replacing H^+^ - nickel aluminate phase (observed also indirectly through the occurrence of high temperature reduction peak during H_2_-TPR analysis, Figure 3). Also, hardly reducible Ni^2+^ species strongly interacting with the zeolite support act as newly generated Lewis acidic sites.

Changes in the nature of acid sites in the Ni/HZSM-5 catalysts after the catalytic hydrogenation reaction show the same trend, regardless of the nickel content (comparison of Figures 5A,B and Table 4). The BAS/LAS ratio increases slightly due to a more notable drop in LAS compared to BAS.

**Figure 6:**
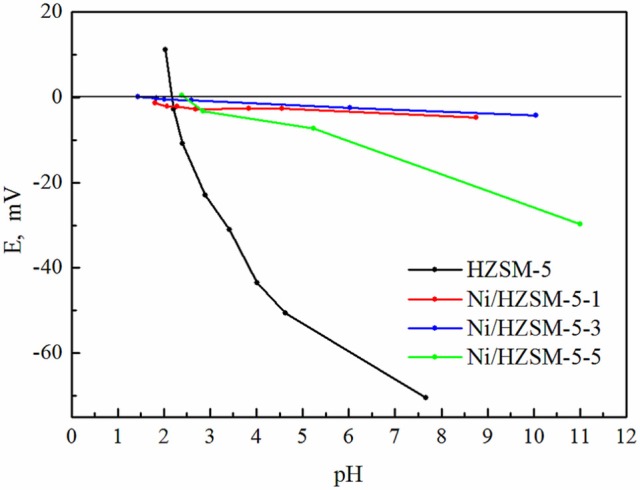
Zeta potencial of HZSM-5 and Ni/HZSM-5 samples with containing 1, 2, and 3 weight % of nickel (Ni/HZSM-5-1, Ni/HZSM-5-3, Ni/HZSM-5-5).

The isoelectric point of the pure HZSM-5 is around 2.5 (Figure 6). With the impregnation of nickel, the isoelectric point of Ni/HZSM-5 samples does not change substantially, since the acidity of their surface is related to the number of BAS which are governed by the amount of framework aluminum. However, the displacement of the isoelectric points for Ni/HZSM-5-1 and Ni/HZSM-5-3 below 2.5 (isoelectric point for HZSM-5, Figure 6) and below 3.5-4 (isoelectric point for NiO, Hernandez et al., 2005) is observed (Figure 5). This means that the Ni and Al interactions in samples with a molar ratio of Ni/Si = 0.01 and 0.03 (Ni/Al < 0.5) can have a higher synergistic effect in reactions than in the sample with a molar ratio of Ni/Si = 0.05 (Ni/Al > 0.5) due to so called spinel like effect (Kosmulski, 2009; Yung et al., 2016). This is confirmed with the fact that the spinel structure is not tolerant concerning the change of Ni/Al molar ratio > 0.5.

**Figure 7:**
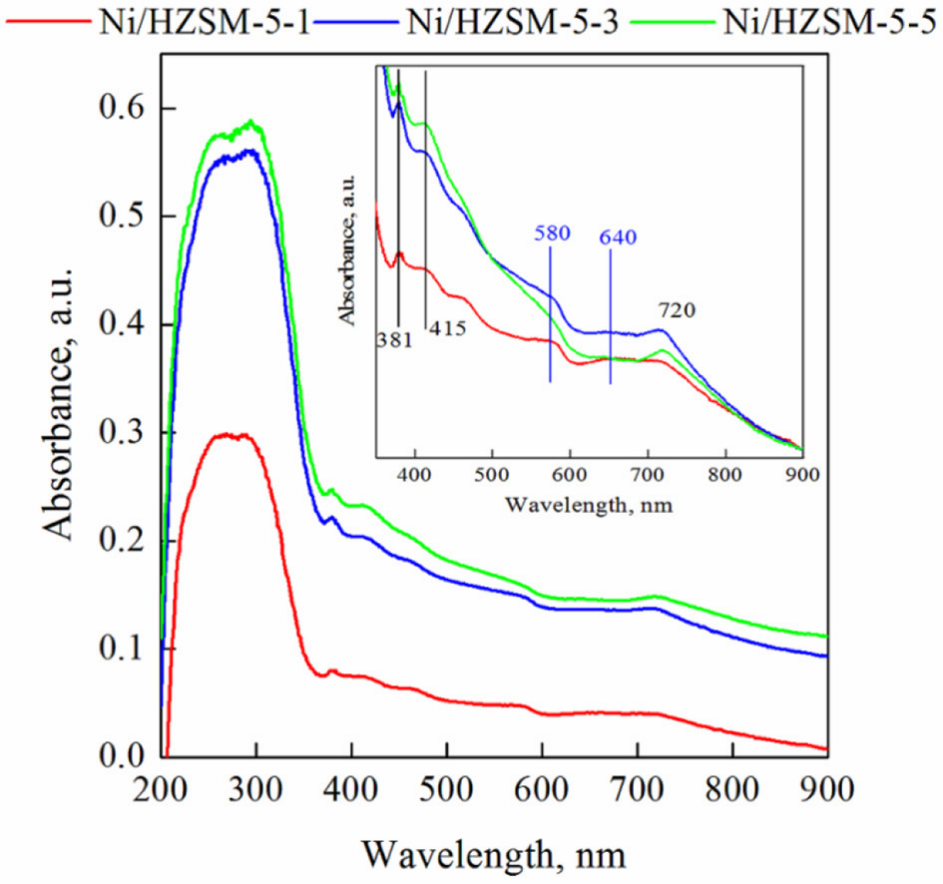
UV-Vis DR spectra of Ni/HZSM-5 catalysts. Inset shows magnification of the range between 350 and 900 nm. The spectra were offset vertically to match intensity at 900 nm for easier comparison.

UV-Vis DRS analysis was performed to probe the electronic transitions and gain more insight into the phases present in the synthesized catalysts. Figure 7 shows that all Ni/HZSM-5 samples absorb light strongly in the UV range (λ < 350 nm), which is associated with O^2−^ → Ni^2+^ ligand to metal charge transfer (LMCT) transition (Pawelec et al., 2004). The bands at 380, 420, and 715 nm are characteristic for octahedrally coordinated Ni^2+^ in NiO lattice (López-Fonseca et al., 2012; Anjaneyulu et al., 2016). It can be seen that all catalysts contain bulk NiO, its contribution is strongest in catalysts containing the highest nickel content, namely Ni/HZSM-5-3 and NiHZSM-5-5. Bands at 580 nm and between 600 and 645 nm are related to the tetrahedrally coordinated Ni^2+^ species in the nickel aluminate phase (Kim et al., 2004). It can be seen that for samples Ni/HZSM-5-1 and Ni/HZSM-5-3 the contribution of nickel aluminate phase is the highest. This is in agreement with the results of H2-TPR and isoelectric point measurements.

**Figure 8:**
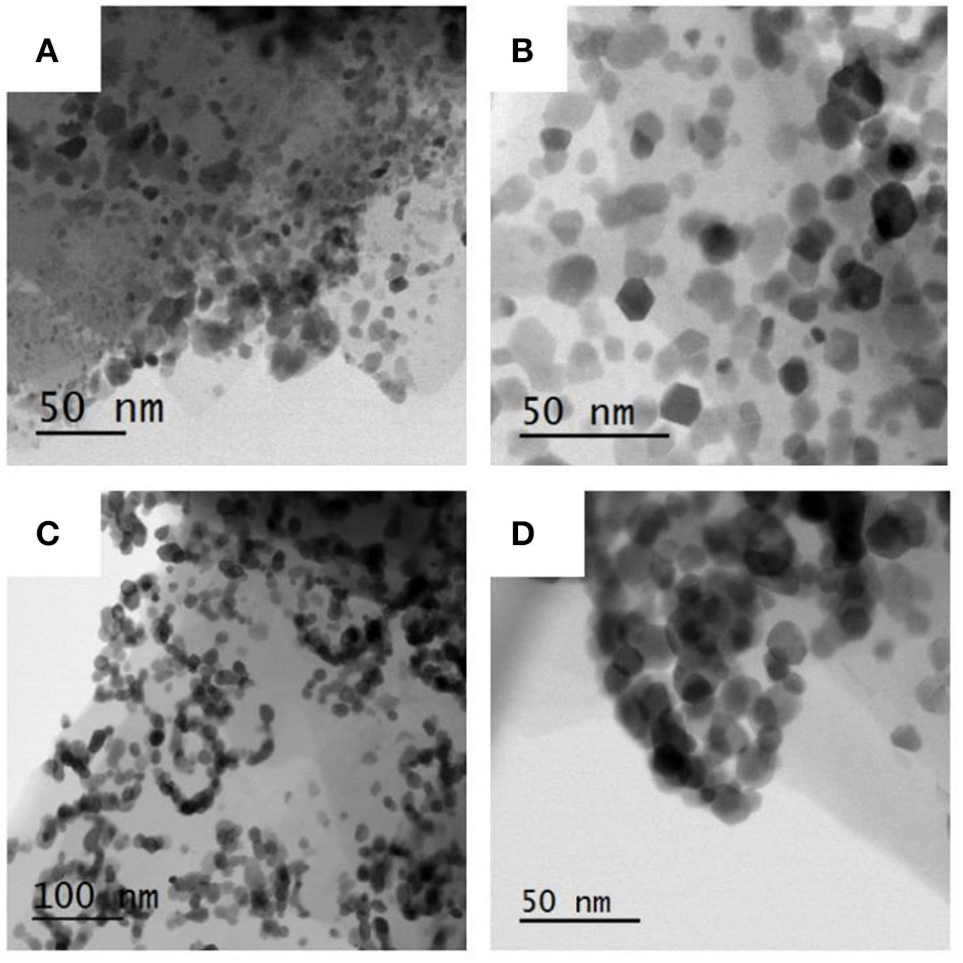
STEM-BF micrographs of NiO particles on zeolite crystallites: **(A)** Ni/HZSM-5-1, **(B)** Ni/HZSM-5-3, **(C, D)** Ni/HZSM-5-5 (note chain-like network of particles).

In Figure 8 STEM-BF micrographs of samples Ni/HZSM-5-1 (Figure 8A), Ni/HZSM-5-3 (Figure 8B) and Ni/HZSM-5-5 (Figures 8C,D) are shown. Regularly shaped NiO particles with nicely expressed crystal planes are distributed uniformly in Ni/HZSM-5-3, and non-uniformly in Ni/HZSM-5-1. In Ni/HZSM-5-5 sample, a specific chain-like network of NiO crystals becomes apparent. The latter suggest presence of NiO phase without direct contact with the HZSM-5 support when the nickel content reaches 2.7 wt. %. The size of the NiO particles in all three samples was between 8 and 30 nm.

**Figure 9:**
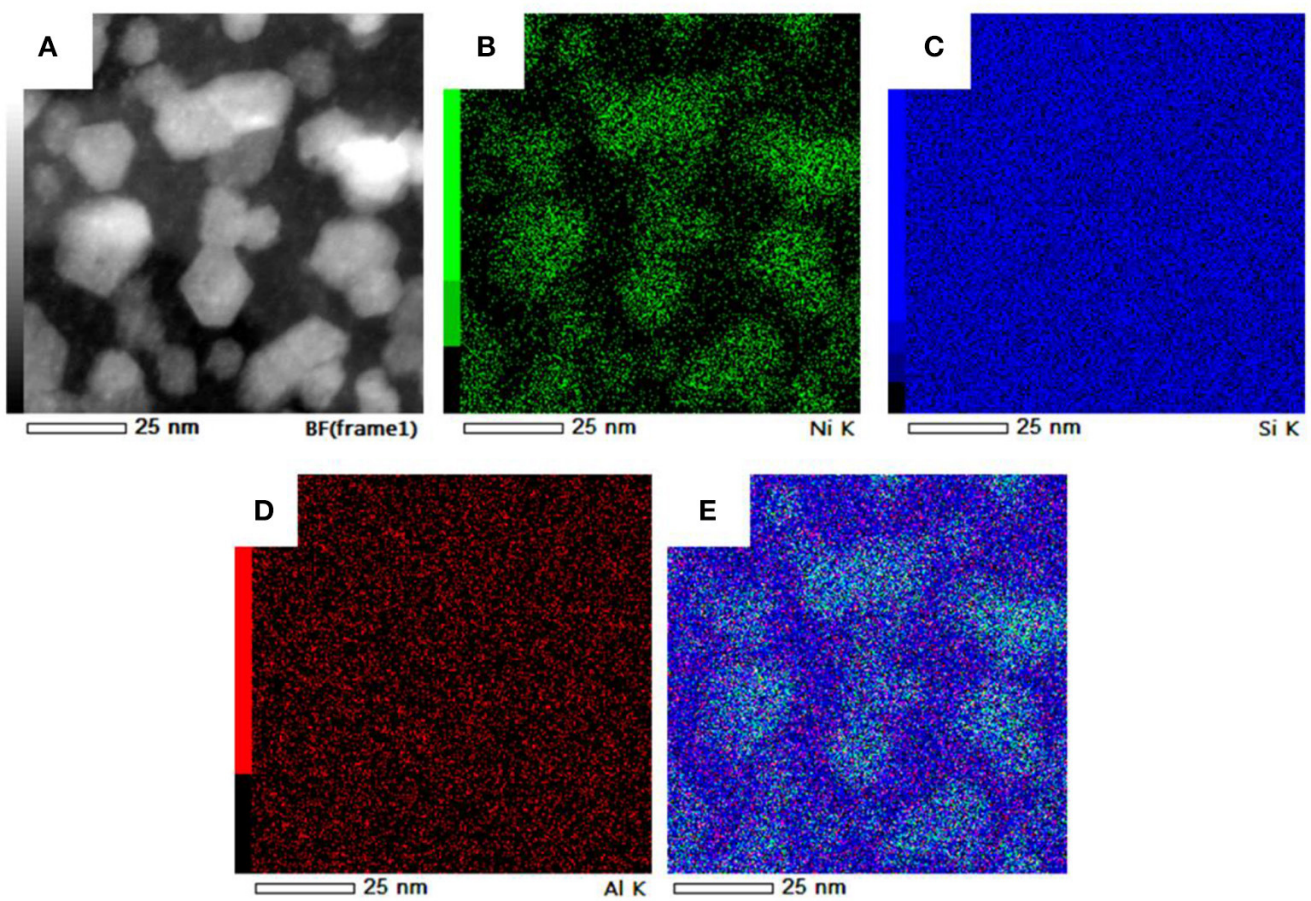
EDXS mapping of support particle in sample Ni/HZSM-5-3 with up to 20 nm sized NiO based particles: **(A)** HAADF micrograph, **(B)** elemental distribution of Ni, **(C)** distribution of Si, **(D)** elemental distribution of Al, **(E)** composite image.

Additional information on distribution of Ni and Al inside the Ni/HZSM-5 catalysts was obtained with TEM-EDXS elemental mapping (Figure 9). Nickel is highly dispersed over the support, in addition to nickel concentrated in NiO particles. This corroborates presence of different nickel containing species, as suggested indirectly by H_2_-TPR and UV-Vis DRS analyses. Aluminum elemental map shows its homogeneous dispersion in the catalyst. This is in agreement with pyridine IR analysis, which suggested that 93% of Al^3+^ is tetrahedrally coordinated and integrated in the zeolite framework.

### Catalytic performance

**Scheme 1:**
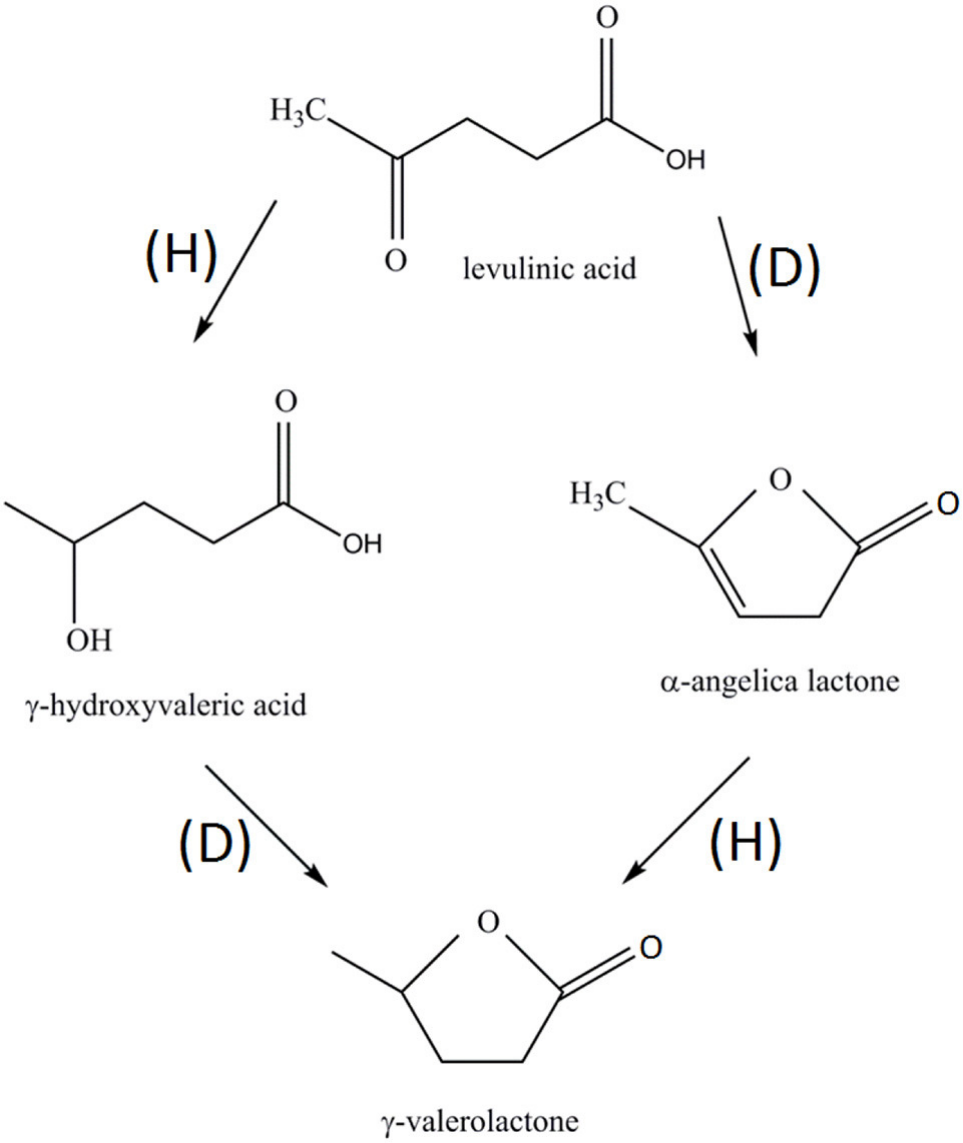
Pathway of catalytic hydrogenation (H) and dehydration (D) reactions involved in the transformation of levulinic acid to GVL.

Catalytic results of the Ni/HZSM-5 catalysts in levulinic acid conversion to GVL are compiled in Table 5. The only reaction products formed in measurable quantities are γ-hydroxyvaleric acid, α-angelica lactone and GVL. GVL can be produced by two independent reaction pathways, which both involve hydrogenation (H) and dehydration (D) reactions (Scheme 1). The order in which reactions (H) and (D) occur is diametrally different in both pathways and depends on the availability and reactivity of active catalytic sites present on the catalysts (acid and metal sites), as well as reaction conditions.

**Table 5:** Catalytic activity and product yields at different reaction temperatures for the studied samples.

**T, ^°^C**	**Catalyst**	**Yield of GVL, wt. %**	**Yield of γ-hydroxyvaleric acid, wt. %**	**Yield of α-angelica lactone, wt.%**	**Conversion, %**
250	ZSM-5-1Ni	18.8	11.7	22.5	52.9
	ZSM-5-3Ni	12.4	21.7	38.0	82.2
	ZSM-5-5Ni	9.0	23.4	45.6	77.9
300	ZSM-5-1Ni	31.4	16.5	3.3	51.2
	ZSM-5-3Ni	92.8	3.5	0	96.3
	ZSM-5-5Ni	90.2	3.2	1.3	94.7
320	ZSM-5-1Ni	57.5	19.3	1.5	78.3
	ZSM-5-3Ni	98.6	0	0	98.6
	ZSM-5-5Ni	95.3	1.4	0	96.7

At 250°C levulinic acid conversions between 53 and 82% were achieved and γ-hydroxyvaleric acid, α-angelica lactone and γ-valerolactone were registered products on all the studied samples. The highest levulinic acid conversion was registered for the Ni/HZSM-5-3 sample (82%). The α-angelica lactone was the predominant product with yields between 22 and 46%, indicating that the dominant reaction is dehydration of levulinic acid over acid sites present at lower reaction temperature. The yield of γ-hydroxyvaleric acid was between 12 and 23% and increased with nickel content. This directly shows that the hydrogenation of levulinic acid becomes increasingly prominent as the hydrogenation function (nickel content) of the catalyst increases. GVL yields were relatively low, between 9 and 19%.

The increase of reaction temperature to 300°C led to a drastic rise in levulinic acid conversion (51–96%) and change in product distribution: GVL became the most abundant reaction product with yields between 31 and 93%. The Ni/HZSM-5 catalyzed the reaction cascade all the way to the ultimate and desired reaction product: GVL. The yields of γ-hydroxyvaleric and α-angelica lactone were below 4%, except for γ-hydroxyvaleric acid on Ni/HZSM-5-1, which remained relatively high at 16%.

No drastic changes in catalysis were observed at 320°C: The Ni/HZSM-5-3 catalyst showed the highest levulinic acid conversion (99%) and 100% GVL yield, α-angelica lactone was produced in yields below 2% on all catalysts. The catalytic activity of Ni/HZSM-5-1 catalyst was markedly lower compared to Ni/HZSM-5-3 and Ni/HZSM-5-5 and γ-hydroxyvaleric acid yield remained relatively high at 19%.

The following paragraphs discuss the role of metallic nickel sites as well as LAS and BAS in the catalytic conversion of levulinic acid to GVL. Metallic catalytic function provided by nickel crystallites is crucial to produce GVL, as it enables hydrogenation of levulinic acid to γ-hydroxyvaleric acid and α-angelica lactone to GVL (Scheme 1). In the synthesized Ni/HZSM-5 catalysts, a notable fraction of nickel (17, 23, and 39% for Ni/HZSM-5-5, Ni/HZSM-5-3 and Ni/HZSM-5-1, respectively, Table 3) exists as poorly reducible Ni^2+^ cations and BAS compensating Ni^2+^ cations (nickel aluminate phase). They are converted to metallic nickel at temperatures (>500°C) much higher compared to reaction temperatures employed here, and are thus very likely present in ionic form with negligible hydrogenation ability. As a result, only a limited amount of nickel remains “free” to form the metallic domains upon reduction. This is most notable in the Ni/HZSM-5-1 sample and as a result of the lagging hydrogenation ability of this catalyst, much lower levulinic acid conversions and GVL yields are achieved compared to Ni/HZSM-5-3 and Ni/HZSM-5-5 catalysts.

The exact discrimination between the individual roles of LAS (electron acceptor) and BAS (proton donor) in the dehydration reactions (γ-hydroxyvaleric acid to GVL and levulinic acid to α-angelica lactone, Scheme 1) is difficult on the tested group of catalysts since they are both present in comparable amounts. Values in Table 4 show that with increasing Ni^2+^ content, the BAS number decreases and LAS number increases. The intrinsic acid strength of LAS is weaker compared to BAS, which was confirmed also with thermogravimetric pyridine desorption tests (Figure 4). The structure of strong BAS in the synthesized HZSM-5 based catalysts is well known from the literature: protons that act as charge compensating ions for the Al^3+^ cations which are tetrahedrally coordinated in the zeolite framework.

The LAS can originate from extra framework Al^3+^ that forms an ill-defined separate AlO_x_ phase (7% of Al^3+^ in pure HZSM-5 was identified as extra framework by pyridine FTIR analysis, Figure 5A and Table 4), which is expected to be present also in Ni/HZSM-5 materials. In addition, ionic Ni^2+^ which are not reduced under reaction conditions, exhibit electrophilic character and consequently Lewis acidity.

The yield of α-angelica lactone at 250°C (primary reaction product formed on acid sites) correlates with total acidity of the catalysts (Ni/HZSM-5-5 > Ni/HZSM-5-3 > Ni/HZSM-5-1) and more specifically, increasing fraction of LAS. As a result, we can conclude that Lewis acid sites are the dominant active sites performing the dehydration function in the cascade of reactions shown in Scheme 1 over Ni/HZSM-5 catalysts. Kumar et al. (2016) came to similar conclusions, stating that a metal site (Ni) in close proximity to a Lewis acid site is active for the selective conversion of LA to GVL.

From above discussion we come to the conclusion that the Brønsted acid sites (nickel aluminate phase) create the synergetic and stabilizing effects of all active sites in the catalyst, when BAS/LAS is approaching to 1 and at the same time the amount of Ni easy reducible active sites is 80% (Ni/HZSM-5-3, Table 4).

**Figure 10:**
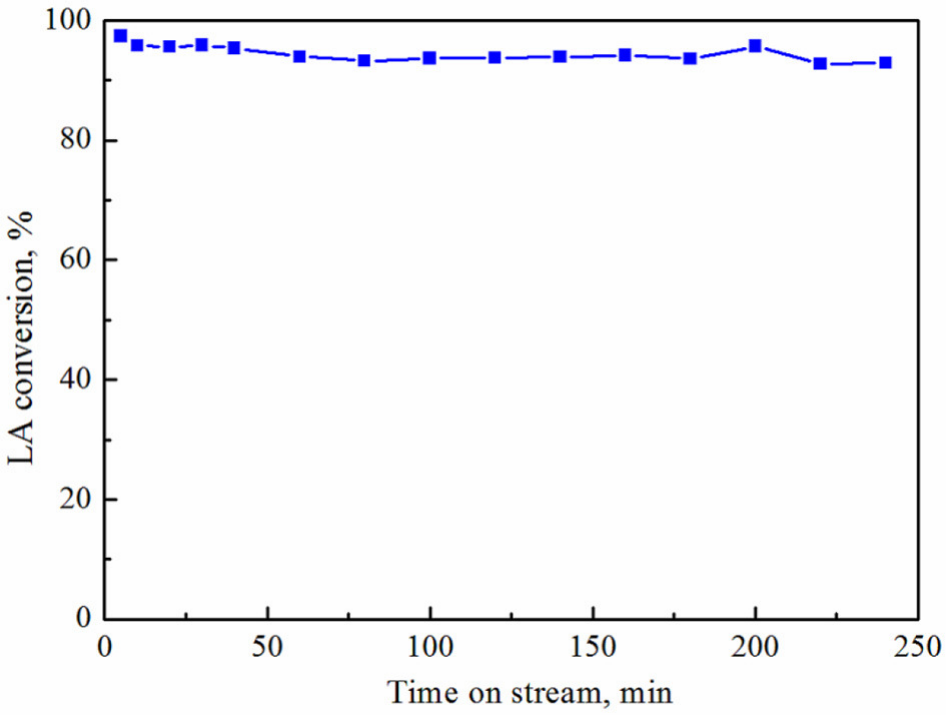
Levulinic acid conversion on Ni/HZSM-5-3 catalyst at 320°C as a function of time on stream.

**Figure 11:**
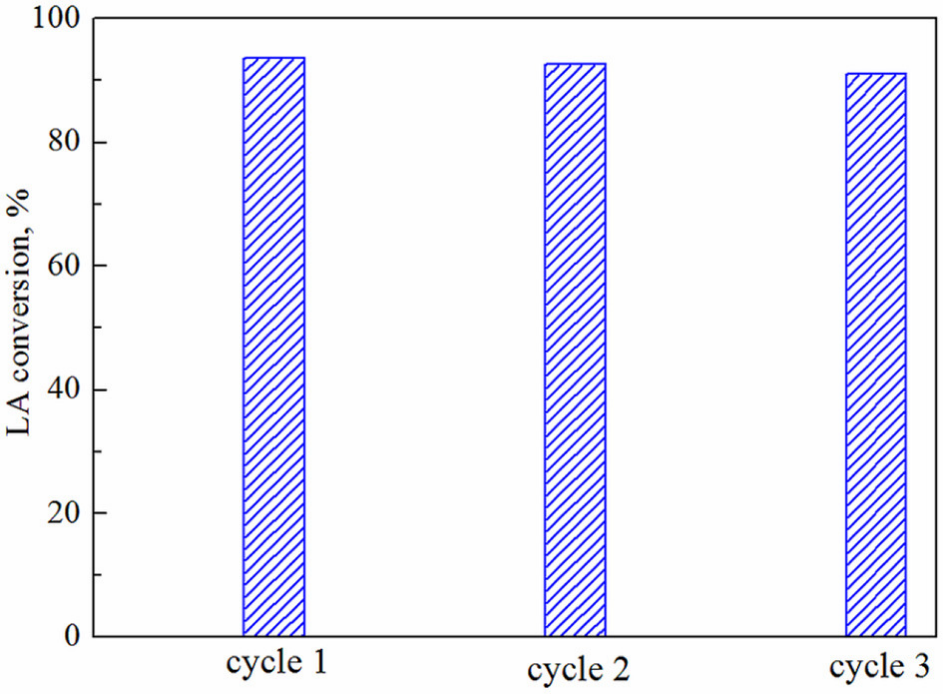
Levulinic acid conversion achieved during three consecutive runs conducted at 320°C using the same batch of Ni/HZSM-5-3 catalyst.

The stability of the Ni/HZSM-5-3 catalyst was studied at 320°C (Figure 10). A negligible decrease in the conversion of levulinic acid was observed during the 240 min of reaction: from 97 to 93.6%. GVL is the only registered product in the studied reaction time in all reaction cycles. After the steady-state stability test, the same catalyst sample was regenerated in air at 500°C, reduced in hydrogen at 400°C, and reexamined in three consecutive reaction cycles at 320°C. During these cycles, only a marginal drop in levulinic acid conversion from 93.6 to 91% was observed (Figure 11). The stability test was performed also at 250°C for 12 h and the small decrease in the activity from 82.2 to 80.5% was registered (not shown). The stable catalytic activity and selectivity to GVL could be explained by sufficient stability of the metallic and acidic functionalities in the synthesized catalysts (under the employed steady state reaction conditions, as well as redox cycling), which makes them a potential candidate for industrial applications.

## Conclusions

HZSM-5 zeolite was synthesized with Al/Si molar ratio of 0.04 (1.7 wt. %) without any organic template and was modified with different Ni content (approx. from 1 to 3 wt. %) by incipient wetness impregnation. TEM-EDX, UV-Vis DRS and H_2_-TPR analyses showed presence of different reducible nickel containing species in the catalysts: (i) bulk crystalline NiO which is the predominant phase in all materials, (ii) Ni^2+^ strongly interacting with the zeolite support and (iii) Ni^2+^ charge compensating cations on Brønsted sites in zeolite. The TPD of pyridine shows that the total amount of acid sites (Lewis and Brønsted) changes very little when Ni content is increased (Ni/HZSM-5-1 < HZSM-5 < Ni/ZSM-5-3 < Ni/HZSM-5-5) and their strength decreases with increasing nickel content: HZSM-5 > Ni/HZSM-5-1 > Ni/HZSM-5-3 > Ni/HZSM-5-5. The latter is due to formation of Lewis acid sites, associated with electrophilic poorly reducible Ni^2+^ species. The fraction of these nickel species is not negligible (17, 23, and 39% for Ni/HZSM-5-5, Ni/HZSM-5-3, and Ni/HZSM-5-1, respectively). They are converted to metallic nickel at temperatures much higher compared to reaction temperatures employed in the present study, and are thus very likely present in ionic form with negligible hydrogenation ability.

Catalytic tests of LA conversion to GVL revealed that the Ni/HZSM-5-3 (Ni/Si = 0.03) catalyst was the most active, and reached 99% conversion of levulinic acid and 100% selectivity to GVL at 320°C. Further increase of Ni content in Ni/HZSM-5-5 (Ni/Si = 0.05) does not lead to improved catalytic activity or selectivity. Ni/HZSM-5-3 catalyst shows also stable levulinic hydrogenation to GVL in 3 reaction cycles carried out at 320°C. The highest catalytic activity and stability of the Ni/HZSM-5-3 catalyst is achieved due to: (1) the metallic hydrogenation function and acidic dehydration have to be present simultaneously in the form of nickel metallic clusters and Lewis acid sites (2) nickel aluminate phase on Brønsted acid sites having synergetic and stability effects on all active sites in the catalysts.

## Author contributions

Synthesis and standard characterization of the catalysts were performed and discussed by AR. Characterization of the catalysts by acidity tests, UV-VIS and IR were performed and discussed by PD and AP. GD performed and discussed TEM characterization. Catalytic tests were performed and discussed by MP, HL, and AB. NT gave the research idea, coordinated the work and led the discussion on the results. All authors listed have made a substantial, direct and intellectual contribution to the work, and approved it for publication.

### Conflict of interest statement

The authors declare that the research was conducted in the absence of any commercial or financial relationships that could be construed as a potential conflict of interest.
